# Comparative RNA sequencing analysis of resistant and susceptible Dendrobium “Earsakul” under black rot challenge

**DOI:** 10.5114/bta/216300

**Published:** 2026-03-25

**Authors:** Piyada A. Tantasawat, Sukanya Inthaisong, Theerawat Chantakot, Kanlayanee Sawangsalee, Apinya Khairum, Pakhawat Pookhamsak, Akkawat Tharapreuksapong, Pakpoom Boonchuen, Sureerat Yenchon, Chunhua Ma

**Affiliations:** 1 School of Crop Production Technology, Institute of Agricultural Technology, Suranaree University of Technology, Nakhon Ratchasima, Thailand; 2 Department of Agricultural Management, Faculty of Interdisciplinary Management and Technology, Kasetsart University, Suphan Buri, Thailand; 3 Department of Horticulture, Faculty of Agriculture, Ubon Ratchathani University, Ubon Ratchathani, Thailand; 4 Center for Scientific and Technological Equipment, Suranaree University of Technology, Nakhon Ratchasima, Thailand; 5 School of Biotechnology, Institute of Agricultural Technology, Suranaree University of Technology, Nakhon Ratchasima, Thailand; 6 Agricultural Innovation and Management Division, Faculty of Natural Resources, Prince of Songkla University, Songkhla, Thailand; 7 College of Landscape and Horticulture, Yunnan Agricultural University, Kunming, China

**Keywords:** *Dendrobium*, defense response-related genes, *Phytophthora parasitica*, resistance mechanisms, RNA sequencing, transcriptomics analysis

## Abstract

**Background:**

*Dendrobium*, a highly popular ornamental plant with immense economic significance, is increasingly threatened by black rot disease caused by *Phytophthora parasitica*. The sole reliance on the use of chemicals to control this disease highlights the need for developing strategies to generate sustainable, genetically resistant *Dendrobium* varieties.

**Materials and methods:**

In this study, we conducted comparative transcriptomic profiling to elucidate the molecular basis of black rot resistance in *Dendrobium* “Earsakul” by analyzing resistant (SUT13E18305) and susceptible (SUT16C007) lines at 0, 12, and 24 h post-inoculation.

**Results:**

Phenotypic evaluation revealed clear divergence between the two lines by 5 days after inoculation (DAI): the resistant line exhibited limited disease symptoms, while the susceptible line showed severe disease progression by 7 DAI. RNA sequencing analysis showed rapid and dynamic defense response in the resistant genotype, including activation of genes encoding pathogen recognition receptors such as chitin elicitor-binding protein-like and receptor-like kinases, which triggered the immune response. Key transcription factor genes (*WRKY43, WRKY75*, and *MYB6*) were strongly upregulated, and these upregulated genes mediated coordinated activation of signaling pathways. Defense-related genes involved in the biosynthesis of pathogenesis-related proteins and antimicrobial compounds, reactive oxygen species management, strengthening of the cell wall, and phenylpropanoid metabolism were also significantly induced in the resistant genotype. In contrast, the susceptible genotype exhibited delayed immune activation, suppressed expression of defense-related genes, and inadequate cell wall remodeling, ultimately leading to extensive colonization of the pathogen. Notably, quantitative real-time polymerase chain reaction confirmed stage-specific expression patterns of multiple genes in both genotypes.

**Conclusions:**

The findings of this study demonstrate the utility of transcriptomics in elucidating complex defense responses in non-model crops and offer broader insights into plant-hemibiotrophic pathogen interactions, providing a molecular framework for breeding disease-resistant *Dendrobium* cultivars.

## Introduction

*Dendrobium*, a major genus within the *Orchidaceae* family, comprises over 200 species and is among the most widely cultivated orchids because of its ease of growth, vibrant floral diversity, and ornamental appeal (Puchooa 2004). Owing to these attributes, *Dendrobium* species are extensively used in landscaping, floral decorations, and export markets. Thailand is currently the world’s leading exporter of tropical orchids, with 2024 export volumes reaching 19,129 tons of cut flowers and 20,597 tons of potted plants, generating revenues of approximately USD 64.52 million and 16.97 million, respectively (Office of Agricultural Economics 2025).

Despite its economic importance, *Dendrobium* production faces a major challenge by black rot, a destructive disease caused by *Phytophthora* spp. – a hemibiotrophic oomycete with both biotrophic and necrotrophic phases (Zhou et al. 2023). This pathogen causes extensive damage across a range of orchid genera, including *Cattleya, Vanda, Phalaenopsis*, and *Dendrobium* (Uchida 1994; Daly et al. 2013; Bag et al. 2024). Infections typically manifest as rapidly progressing water-soaked lesions, ultimately leading to tissue necrosis and plant collapse. Chemical control remains the primary management strategy for black rot disease; however, it poses risks to human health and environmental sustainability, highlighting the need for more durable and eco-friendly solutions.

To combat biotic stress, plants have evolved multilayered defense mechanisms, including morphological barriers, biochemical responses, and molecular signaling networks. The central components underlying these defense systems are resistance (R) genes, which encode proteins that can recognize pathogen-derived molecules (effectors) and activate downstream defense pathways (Wu et al. 2014). R proteins, often localized on the plasma membrane or within the cytoplasm, initiate a cascade of immune responses following effector recognition, including hypersensitive response (HR), production of antimicrobial compounds (e.g., phytoalexins), cell wall remodeling, and systemic acquired resistance (SAR) (Eckardt 2007; Ding et al. 2022). R genes have been implicated in resistance to a broad range of pathogens such as *Magnaporthe oryzae* in rice (Khan et al. 2018), *Xanthomonas* spp. in *Arabidopsis* and wheat (Liu et al. 2017; Peng et al. 2019), and *Erwinia amylovora* in apple (Oh and Beer 2007; Campa et al. 2019).

Breeding for disease resistance is a key tenet of sustainable crop improvement. Integrating R genes into elite cultivars through molecular breeding and marker-assisted selection enhances resistance durability (Valliyodan et al. 2017). In this context, transcriptome profiling offers a robust tool to elucidate complex plant–pathogen interactions. RNA sequencing (RNA-Seq) provides comprehensive insights into gene expression dynamics during host–pathogen interactions and has been widely used to clarify resistance-related pathways. For example, transcriptome profiling has revealed resistance and susceptibility genes in tobacco cultivars challenged with *Phytophthora nicotianae* (Meng et al. 2024), clarified defense responses in melon against *P. capsici* (Wang et al. 2020), and demonstrated the synergistic activation of salicylic acid (SA) and jasmonic acid (JA) signaling in *Arabidopsis thaliana* (Zhang et al. 2020). Given the limited genomic information available for orchids, particularly regarding defense mechanisms, transcriptomic studies are vital for understanding the responses of host orchid plants to *P. parasitica*. Therefore, the present study aimed to investigate the transcriptional responses of black rot-resistant and black rot-susceptible lines of *Dendrobium* “Earsakul” at 0, 12, and 24 h post-inoculation (hpi) by using RNA-Seq analysis. The identification of differentially expressed genes (DEGs) and the associated defense pathways provides a foundation for developing molecular markers and breeding strategies for durable resistance in orchids.

## Materials and methods

### Plantlet materials

**Figure 1:**
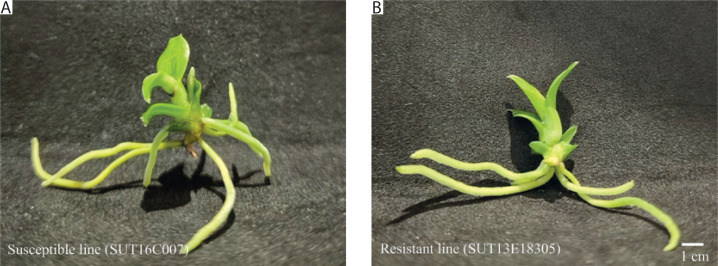
Morphological characteristics of the susceptible line SUT16C007 (A) and the resistant line SUT13E18305 (B) at 8 months of age in culture media

In our previous study, two contrasting lines of *Dendrobium* “Earsakul” were developed for evaluating black rot resistance: a wild-type, non-mutagenized susceptible line, SUT16C007, and an ethyl methanesulfonate-mutagenized line, SUT13E18305, which exhibited high resistance to *P. parasitica* (Khairum et al. 2022). The morphological characteristics of both lines are shown in Figure 1. In the present study, these genotypes were selected to investigate host–pathogen interactions and transcriptional responses to *P. parasitica* infection. Prior to inoculation, plantlets were propagated on modified Vacin and Went medium (Vacin and Went 1949; Tantasawat et al. 2015; Khairum et al. 2022) and subsequently transplanted to coconut husk substrate at the age of 8 months.

### Evaluation of black rot resistance with a detached leaf assay

Both resistant (SUT13E18305) and susceptible (SUT16C007) *Dendrobium* “Earsakul” plantlets were cultivated in coconut husk under greenhouse conditions. At 6 months post-transplantation, the plantlets were in the vegetative stage with 4–5 leaves, were successfully acclimatized, and showed active leaf and root development. The leaves were inoculated with *P. parasitica* by placing a 4-mm mycelial disk at the tip of each leaflet at node 2. One disk was applied per leaflet per plantlet, with three biological replicates (one plantlet per replicate per genotype) maintained under controlled laboratory conditions (16-h light/8-h dark photoperiod), following a modified protocol of Khairum et al. (2022). Disease severity was assessed at 0, 1, 3, 5, and 7 days after inoculation (DAI) and classified into five categories based on symptom progression: highly resistant (HR; 0.00–0.49), resistant (R; 0.50–1.49), moderately resistant (MR; 1.50–2.49), moderately susceptible (MS; 2.50–3.49), and susceptible (S; 3.50–5.00), following the rating scale described by Khairum et al. (2022). The experiment was arranged in a completely randomized design. Disease severity scores were analyzed using SPSS version 16.0. Statistical significance was determined by an *F*-test, and a *p*-value of ≤ 0.05 was considered statistically significant.

### mRNA preparation, cDNA library construction, and sequencing

The leaves of susceptible and resistant *Dendrobium* “Earsakul” lines were cultivated and inoculated using the same procedure as described above. Leaf samples were then collected at 0, 12, and 24 hpi for RNA extraction using the FavorPrep Plant Total RNA Mini Kit (Favorgen Biotech Corp., Pingtung, Taiwan), in accordance with the manufacturer’s instructions (Li et al. 2021; Wang et al. 2020). RNA concentration and purity were measured using a Nanodrop spectrophotometer, and RNA integrity was confirmed with 1% agarose gel electrophoresis to ensure sample quality and prevent degradation. Next, 1 µg of total RNA was used for cDNA library preparation; the prepared library was sequenced on an Illumina HiSeq, NovaSeq6000, or MGISEQ-2000 instrument with a 2 × 150 paired-end configuration.

### Transcriptome profiling and functional interpretation of gene expression

The original sequence data (pass filter data) were subjected to quality control check and then used for bioinformatics analysis. To ensure data quality, low-quality reads and errors were removed using Cutadapt (v1.9.1) software (Martin 2011). The sequenced reads were mapped to the *Dendrobium catenatum* reference genome (NCBI RefSeq assembly: GCF_001605985.2, https://www.ncbi.nlm.nih.gov/datasets/genome/GCF_001605985.2/) using Hisat2 (v2.2.1) (Kim et al. 2015). The full-length transcripts from the aligned reads were generated using a GFF file and subsequently indexed. HTSeq (v0.6.1) was used to estimate gene and isoform expression levels from the paired-end clean data. Differential expression was analyzed with DESeq2, and significant genes were identified based on the following criteria: fold change ≥ 2 and *q*-value (FDR, *p* adj) < 0.05 (Love et al. 2014). DEGs are the key genes for identifying different biological processes and responses to various treatments.

### Gene Ontology (GO) analysis and Kyoto Encyclopedia of Genes and Genomes (KEGG) pathway enrichment analysis

GO analysis and KEGG pathway enrichment analysis are widely used methods for interpreting transcriptomic data. GO terms provide a framework for categorizing enriched genes based on their biological processes (BP), molecular functions (MF), and cellular components (CC) and offer a comprehensive understanding of their functional roles. GOSeq (v1.34.1) was used to identify GO terms of annotated genes with significance considered at *p* adj ≤ 0.05 (Harris 2004; Young et al. 2010). KEGG, a collection of databases, maps gene expression to genomes, biological pathways, diseases, and chemical substances. The in-house scripts were used to determine the enrichment of significant DEGs in KEGG pathways (Kanehisa et al. 2000). In this experiment, the results of DEGs were used to explore key metabolic and signaling pathways associated with resistance-related genes.

### Annotated gene prediction and validation through quantitative real-time polymerase chain reaction (qPCR)

RNA-Seq analysis was used to validate and select raw reads based on significant differences in expression levels. Primers for candidate genes were designed using Primer3Plus software (Supplementary Table 1). For cDNA synthesis, 1 µg of total RNA was reverse transcribed with the iScript Reverse Transcription Supermix Kit (Bio-Rad Laboratories, Hercules, CA, USA). qPCR amplification was performed with 1× Luna Universal qPCR Mix (New England Biolabs Inc., Ipswich, MA, USA), 0.1 pM of each specific primer, and 1 µg of cDNA. The conditions were as follows: initial denaturation at 95°C for 2 min, followed by 40 cycles of denaturation at 95°C for 5 s, annealing at 60°C for 30 s, and extension at 60°C for 5 s (Jaree et al. 2022). In qPCR, the cycle threshold (Ct) represents the cycle where the fluorescence signal crosses a set threshold, indicating gene amplification. Ct values were normalized against a stable reference gene (actin gene) and were averaged across three biological replicates to determine relative mRNA expression levels using the comparative Ct (2^–ΔΔCT^) method with 2–3 technical replicates. Statistical analysis of relative expression levels was conducted using SPSS version 16.0, with a significant *F*-test result defined as *p* ≤ 0.05. The correlation between RNA-Seq and qPCR results was evaluated by Pearson’s correlation analysis using SPSS version 16.0.

## Results

### Evaluation of differential responses to black rot infection in resistant and susceptible Dendrobium lines

**Figure 2:**
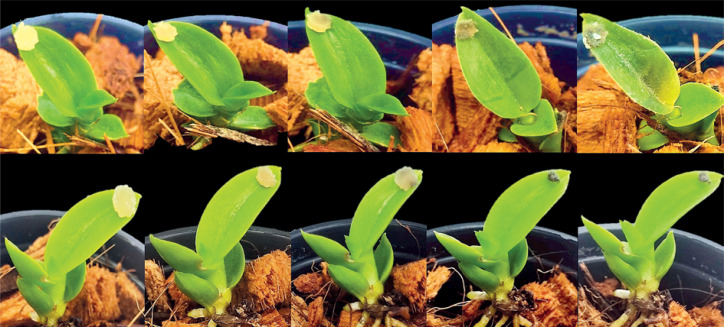
Black rot symptoms in the susceptible and resistant lines of Dendrobium “Earsakul” following Phytophthora parasitica infection as determined by detached leaf assay at 0, 1, 3, 5, and 7 days after inoculation

The severity of black rot disease was compared between the SUT16C007 susceptible line and the SUT13E18305 resistant line following *P. parasitica* inoculation. Disease progression was monitored at 0, 1, 3, 5, and 7 DAI (Table 1; Figure 2). The two genotypes showed no significant differences in disease severity scores at 0, 1, and 3 DAI (*p* > 0.05), although the resistant line began to show a trend toward lower disease severity by 3 DAI. A significant difference between the two genotypes emerged at 5 DAI (*p* < 0.05), when the susceptible line exhibited a severity score of 3.33 and was classified as MS, while the resistant line showed minimal symptoms with a score of 0.33 and was classified as HR. At this time point, the susceptible line exhibited initial black rot lesions on its leaves, whereas the resistant line remained largely asymptomatic.

**Table 1:** Black rot disease severity scores of resistant and susceptible lines of *Dendrobium* “Earsakul” assessed by detached leaf assay at 0, 1, 3, 5 and 7 days after inoculation (DAI)

Lines	Disease severity score on different DAI									
	0 DAI		1 DAI		3 DAI		5 DAI		7 DAI	
Susceptible line SUT16C007	0.00 ± 0.00^2^	HR^3^	0.00 ± 0.00	HR	1.67 ± 0.54	MR	3.33 ± 0.68^a^	MS	4.00 ± 0.47^a^	S
Resistant line SUT13E18305	0.00 ± 0.00	HR	0.00 ± 0.00	HR	0.00 ± 0.00	HR	0.33 ± 0.27^b^	HR	0.67 ± 0.27^b^	R
*F*-test^1^	NS		NS		NS		*		**	

1 NS, *, ** indicates non-significant, a significant difference between lines at each infection time at *p* < 0.05 and *p* < 0.01, respectively.

2 Mean ± SE in the same column with different letters are significantly different (*p* < 0.05) based on Duncan’s Multiple Range Test (DMRT).

3 Disease response refers to black rot resistance levels based on severity scores were as follow: highly resistant (HR) = 0.00–0.49, resistant (R) = 0.50–1.49, moderately resistant (MR) = 1.50–2.49, moderately susceptible (MS) = 2.50–3.49, and susceptible (S) = 3.50–5.00.

By 7 DAI, the two genotypes showed further differences in disease severity. The SUT16C007 susceptible line (classified as S) exhibited a significant increase in severity (score: 4.00), accompanied by widespread development of lesions and visible fungal mycelium around infection sites. In contrast, the SUT13E18305 resistant line (classified as R) maintained a low severity score of 0.67 (*p* < 0.01) and showed no apparent disease symptoms, with leaves remaining green and healthy. These findings demonstrate that differential disease responses between resistant and susceptible lines can be detected as early as 3 DAI, with prominent and significant differences evident from 5 DAI onward. The contrasting phenotypic responses suggest distinct underlying defense mechanisms contributing to black rot resistance in *Dendrobium* “Earsakul.”

### Transcriptomic profiling of the resistant and susceptible Dendrobium lines in response to P. parasitica infection

RNA-Seq analysis was performed on 18 libraries derived from two *Dendrobium* genotypes, SUT16C007 and SUT13E18305, at various time points following *P. parasitica* infection. Leaf samples were collected at 0, 12, and 24 hpi, with three biological replicates per time point. Sequencing was conducted using the Illumina platform, and high-quality sequence data were obtained, as evidenced by > 90% of bases achieving a Q30 score. After quality filtering, a total of 836.9 million clean reads were retained from an initial 849.8 million raw reads. Individual libraries contained between 40.4 and 52.8 million reads, with clean read lengths ranging from 144.3 to 147.7 bp (Table 2). Gene expression quantification was performed using HT-seq (v 0.6.1), with transcript abundance normalized as fragments per kilobase of transcript per million mapped reads. This approach enabled systematic identification of DEGs associated with the contrasting immune responses of the resistant and susceptible *Dendrobium* “Earsakul” lines to *P. parasitica* infection.

**Table 2:** Raw and clean sequence details of resistant and susceptible *Dendrobium* genotypes under *Phytophthora parasitica* inoculated at 0, 12 and 24 hpi

Samples	Total sequences		Sequence length		Q30 (%)	
	Raw read	Clean read	Raw read	Clean read	Raw read	Clean read
SUT16C007 (0 hpi)-1	40,843,824	40,474,986	150.00	144.99	92.48	92.87
SUT16C007 (0 hpi)-2	44,899,600	44,463,986	150.00	144.98	92.75	93.11
SUT16C007 (0 hpi)-3	41,332,650	40,971,864	150.00	144.30	92.61	92.98
SUT16C007 (12 hpi)-1	42,926,220	42,103,006	150.00	147.72	94.12	95.49
SUT16C007 (12 hpi)-2	53,834,646	52,870,630	150.00	147.71	94.16	95.50
SUT16C007 (12 hpi)-3	47,542,386	46,900,410	150.00	147.99	94.25	95.25
SUT16C007 (24 hpi)-1	50,751,140	50,070,294	150.00	147.85	94.39	95.39
SUT16C007 (24 hpi)-2	45,788,932	44,965,854	150.00	147.61	94.09	95.44
SUT16C007 (24 hpi)-3	53,757,328	52,732,920	150.00	147.56	93.95	95.37
SUT13E18305 (0 hpi)-1	46,560,080	46,097,250	150.00	145.56	92.25	92.56
SUT13E18305 (0 hpi)-2	44,213,792	43,763,952	150.00	144.26	92.52	92.91
SUT13E18305 (0 hpi)-3	41,803,042	41,330,098	150.00	144.82	92.33	92.72
SUT13E18305 (12 hpi)-1	45,052,870	44,349,766	150.00	147.73	94.39	95.60
SUT13E18305 (12 hpi)-2	51,178,812	50,254,396	150.00	147.68	94.31	95.63
SUT13E18305 (12 hpi)-3	53,232,892	52,008,794	150.00	147.22	93.93	95.51
SUT13E18305 (24 hpi)-1	54,004,346	52,893,482	150.00	147.42	93.92	95.43
SUT13E18305 (24 hpi)-2	48,055,278	47,434,244	150.00	147.73	94.33	95.25
SUT13E18305 (24 hpi)-3	44,010,356	43,222,128	150.00	147.64	94.27	95.60
Total	849,788,194	836,908,060	–	–	–	–

**Figure 3:**
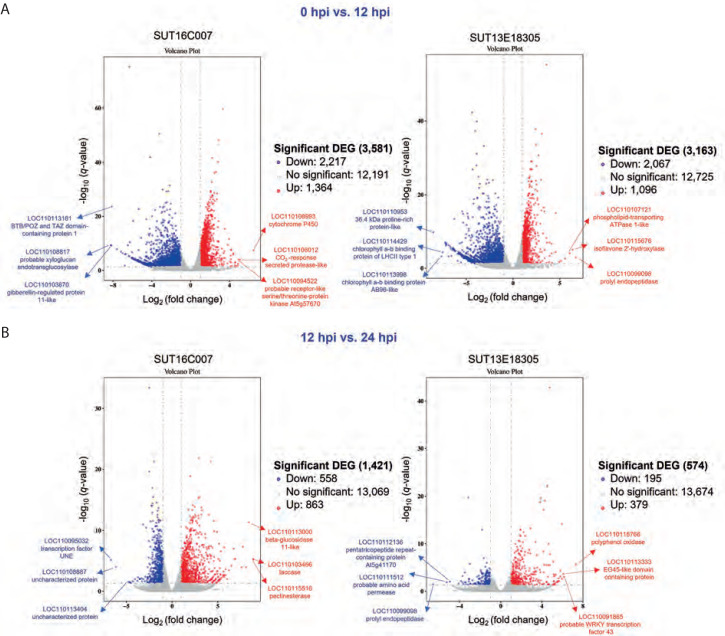
Volcano plot showing differentially expressed genes (DEGs) in the resistant (SUT13E18305) and susceptible (SUT16C007) genotypes at 0 hpi vs. 12 hpi (A) and 12 hpi vs. 24 hpi (B) conditions. Blue dots denote downregulated DEGs, while red dots indicate upregulated DEGs. The X-axis represents the log2 fold change in gene expression, and the Y-axis showsstatistical significance of differential expression, expressed as log10 (q value (FDR, p adj.))

This investigation prioritized the identification of induced DEGs potentially associated with the mechanisms underlying black rot resistance in *Dendrobium* spp. We conducted targeted analysis of plant defense-related genes implicated in the resistance response and employed DESeq2 to determine significant transcriptional changes based on the following stringent thresholds: fold change > 2 and q-value [FDR] < 0.05. Volcano plot visualization (Figure 3) effectively captured the temporal dynamics of DEG distribution across genotypes during infection progression. Comparative transcriptomic profiling between the resistant and susceptible lines revealed distinct temporal response patterns. During the early stage of infection (0 hpi vs. 12 hpi), the susceptible line exhibited 3581 DEGs (2217 downregulated; 1364 upregulated), while the resistant line showed 3163 DEGs (2067 downregulated; 1096 upregulated). The later infection stages (12 hpi vs. 24 hpi) demonstrated substantial response attenuation, with the susceptible line displaying 1421 DEGs (558 downregulated; 863 upregulated) and the resistant line showing only 574 DEGs (195 downregulated; 379 upregulated). Notably, no overlap in gene IDs was observed between conditions within each genotype, emphasizing the temporal specificity of defense responses. These findings delineate the dynamic transcriptional reprogramming occurring during *P. parasitica* infection, particularly related to the quantitative and temporal differences between resistant and susceptible phenotypes. During the early infection stage, the top 3 upregulated genes in the susceptible line were those encoding cytochrome P450, a CO_2_-responsive secreted protease, and the probable receptor-like serine/threonine-protein kinase At5g57670. Conversely, the top 3 most significantly downregulated genes were those encoding BTB/POZ and TAZ domain-containing protein 1, a probable xyloglucan endotransglucosylase, and a gibberellin-regulated protein 11-like protein. In the resistant line, the top 3 upregulated genes were those encoding prolyl endopeptidase, isoflavone 2′-hydroxylase, and a phospholipid-transporting ATPase 1-like protein. The top 3 most strongly downregulated genes were those encoding a 36.4-kDa proline-rich protein, chlorophyll a-b binding protein of LHCII type 1, and a chlorophyll a-b binding protein AB96-like. In the later infection stage, the susceptible line showed highest upregulation of genes encoding laccase, pectinesterase, and beta-glucosidase 11-like, while the gene encoding for the transcription factor UNE12 was the most downregulated one. In the resistant line, the most significantly upregulated genes encoded for polyphenol oxidase, a probable WRKY transcription factor 43, and an EG45-like domain-containing protein, while the top 3 significantly downregulated genes were those encoding for prolyl endopeptidase, a probable amino acid permease, and the pentatricopeptide repeat-containing protein At5g41170 (Figure 3). Thus, the identified DEG repertoire provides a valuable resource for elucidating molecular determinants of black rot resistance in *Dendrobium*.

### GO analysis and KEGG pathway enrichment analysis

**Figure 4:**
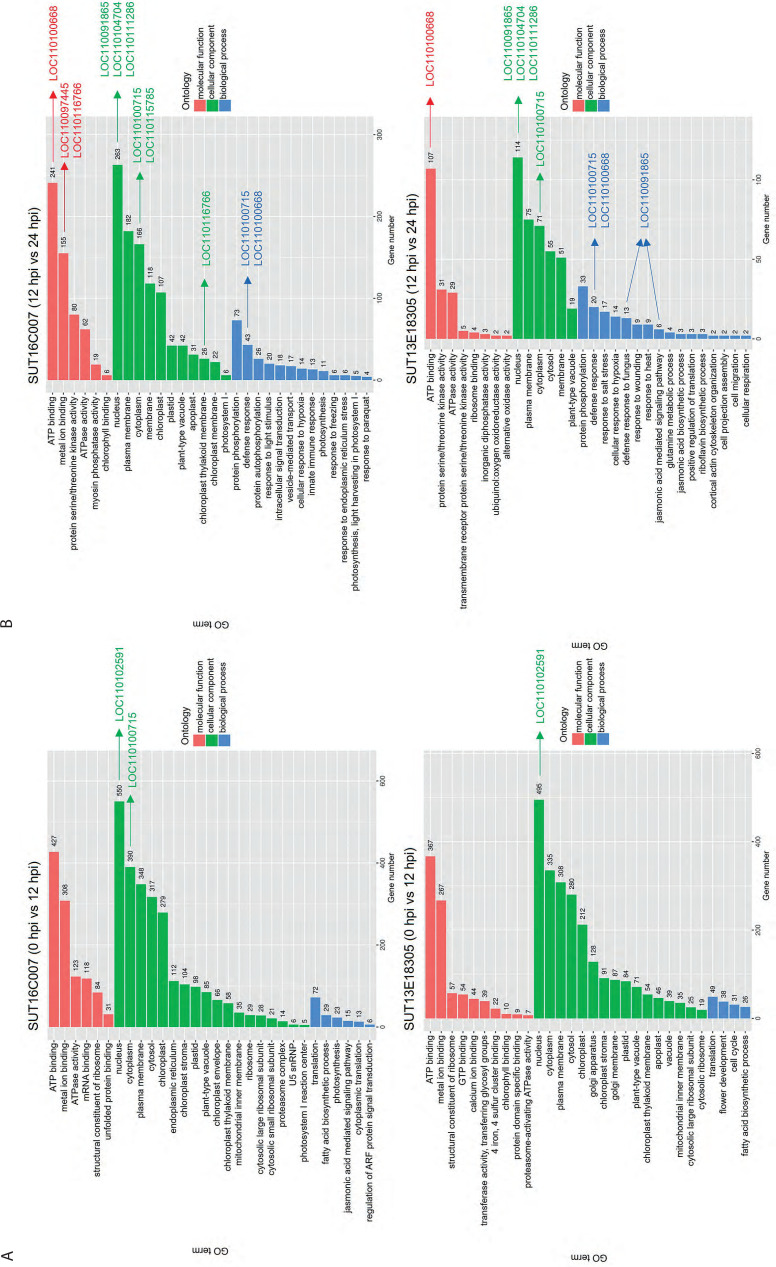
GO categorization of the DEGs in the resistant (SUT13E18305) and susceptible (SUT16C007) Dendrobium lines at 0 hpi vs. 12 hpi (A) and 12 hpi vs. 24 hpi (B), showing significantly enriched BP, CC, and MF term.

DEGs associated with plant defense responses following infection were identified at critical time points (12 and 24 hpi). GO categories were classified into three main groups: BP, MF, and CC. The results showed enriched GO terms in both susceptible and resistant lines, with 0 hpi vs. 12 hpi representing the early response stage and 12 hpi vs. 24 hpi representing the later response stage (Figure 4). In the susceptible line, DEGs in the early response stage were significantly enriched in 1091 MF, 2545 CC, and 158 BP terms. In the later response stage, the DEGs were significantly enriched in 563 MF, 1005 CC, and 256 BP terms. In the resistant line, DEGs in the early response stage were significantly linked with 876 MF, 2309 CC, and 144 BP terms, whereas in the later response stage, the DEGs were significantly associated with 183 MF, 385 CC, and 142 BP terms.

Interestingly, in the later response stage, BP terms in both lines revealed various candidate resistance-related genes. In the susceptible line, several key defense-related processes were significantly enriched, including protein phosphorylation (73 DEGs), defense response (43 DEGs), protein autophosphorylation (26 DEGs), response to light stimulus (20 DEGs), intercellular signal transduction (18 DEGs), vesicle-mediated transport (17 DEGs), cellular response to hypoxia (14 DEGs), innate immune response (13 DEGs), response to freezing (6 DEGs), and response to endoplasmic reticulum stress (6 DEGs). In the resistant line, DEGs in the later response stage were also associated with several key BP-related defense and stress responses, such as protein phosphorylation (33 DEGs), defense response (20 DEGs), response to salt stress (17 DEGs), cellular response to hypoxia (14 DEGs), defense response to fungus (13 DEGs), response to wounding (9 DEGs), response to heat (9 DEGs), JA-mediated signaling pathway (6 DEGs), glutamine metabolic process (4 DEGs), and JA biosynthetic process (3 DEGs).

In the later stage (12 hpi vs. 24 hpi), both susceptible and resistant lines exhibited enrichment of general defense-related BP terms. However, the resistant line demonstrated additional activation of more specific defense pathways, including JA signaling; defense response to fungal pathogens; and responses to abiotic stresses such as salt, heat, and wounding. These pathways likely represent key candidate mechanisms underlying resistance to *P. parasitica* infection. Several interesting genes with high expression levels were identified (Figure 4).

**Figure 5:**
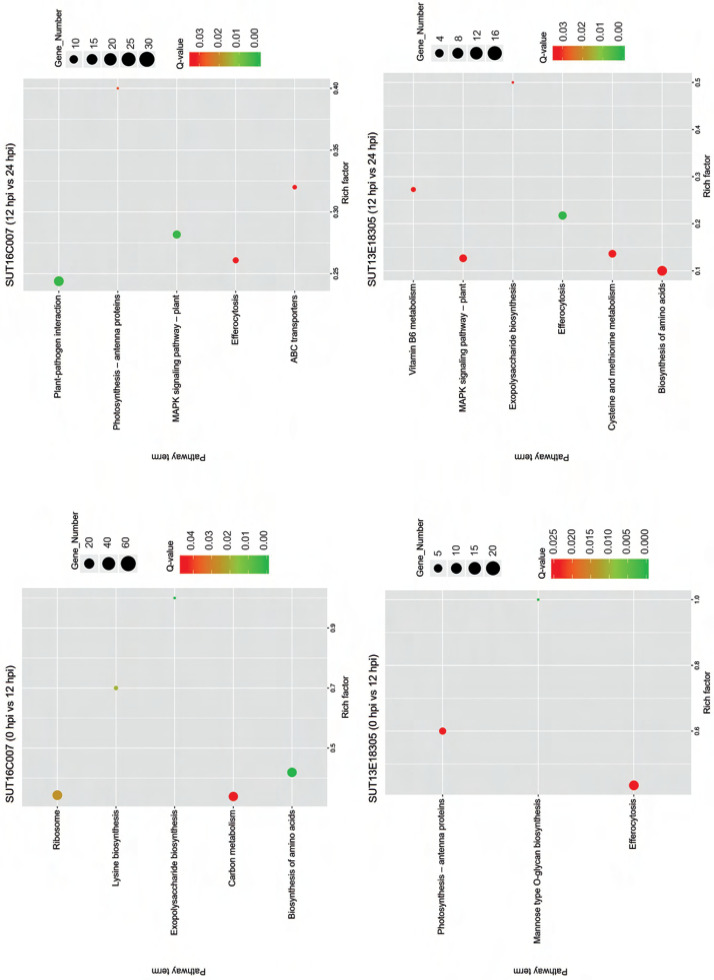
KEGG pathways with significantly enriched differentially expressed genes (DEGs) in the early response stage (0 hpi vs. 12 hpi) and the late response stage (12 hpi vs. 24 hpi) in the resistant (SUT13E18305) and susceptible (SUT16C007) genotypes. The circle area indicates the number of enriched DEGs, and the circle color indicates the range of q-value. Rich factor refers to the ratio of the number of genes differentially expressed in the pathway to the total number of genes in the pathway. Q value is the p value after multiple hypothesis testing and ranges between 0 and 1; the closer the Q value to zero, the more significant is the enrichment.

The KEGG pathway enrichment analysis revealed the significant biological pathways associated with specific DEGs (Figure 5). During early infection, the significantly enriched pathways in the susceptible line were carbon metabolism (46 upregulated genes; 22 downregulated genes), ribosome (13 upregulated genes; 61 downregulated genes), lysine biosynthesis (2 upregulated genes; 5 downregulated genes), biosynthesis of amino acids (48 upregulated genes; 19 downregulated genes), and exopolysaccharide biosynthesis (2 specific downregulated genes), molecular basis of plant growth and developmental processes. In the later infection stage, all 6 photosynthesis-related DEGs were downregulated in the susceptible line. Transport mechanisms showed differential regulation, with ABC transporters exhibiting 5 upregulated and 3 downregulated DEGs (ABC transporter A and B family members), while efferocytosis pathways contained 10 upregulated and 2 downregulated genes. Notably, plant–pathogen interaction pathways revealed 28 upregulated DEGs, including the genes encoding WRKY transcription factors (TFs), Pto-interacting proteins, and calmodulins, and 2 downregulated genes (heat shock protein and 3-ketoacyl-CoA synthase). The MAPK signaling pathway showed pronounced gene activation, with 18 upregulated DEGs (such as genes encoding mitogen-activated protein kinase, serine/threonine-protein kinase, and WRKY TFs), but only 2 downregulated DEGs.

During early infection, the efferocytosis pathway in the resistant line exhibited 15 upregulated and 5 downregulated DEGs. Arabinosyl transferase, a key enzyme in mannose-type O-glycan biosynthesis, showed specific upregulation in the resistant genotype. Conversely, all 9 DEGs encoding chlorophyll a-b binding proteins were completely downregulated in photosynthetic pathways. During the later infection stage, we identified DEGs associated with six key pathways, including vitamin B_6_ metabolism (3 upregulated genes), exopolysaccharide biosynthesis (representing an upregulated gene encoding probable serine acetyltransferase), efferocytosis (8 upregulated and 2 downregulated DEGs), cysteine and methionine metabolism (8 upregulated and 1 downregulated DEGs), amino acid biosynthesis (15 upregulated and 1 downregulated DEGs), and MAPK signaling pathway (8 upregulated and 1 downregulated DEGs). Notably, the majority of these DEGs were upregulated. A particular interesting finding is that 8 upregulated DEGs in the MAPK signaling pathway in the resistant line encoded mitogen-activated protein kinase kinase, serine/threonine-protein kinase, protein phosphatase, transcription factor MYC2, WRKY transcription factor, and ethylene receptor. All identified genes showed significant transcriptional activation following pathogen challenge, suggesting their potential functional involvement in black rot resistance mechanisms.

### Identification of putative genes involved in black rot disease resistance in Dendrobium

We conducted comparative transcriptomics analysis to identify putative black rot disease resistance genes in *Dendrobium* based on the evaluation of DEGs across the susceptible and resistant genotypes during early (0 hpi vs. 12 hpi) and later (12 vs. 24 hpi) infection stages. DEGs at various infection stages are summarized in Supplementary Tables 2 and 3.

### Early-stage transcriptomic responses to P. parasitica infection in Dendrobium genotypes

In the early stage of *P. parasitica* infection, DEGs were identified across multiple functional categories, including pathogen recognition and signal transduction, hormone signaling pathways, transcriptional regulation, PR proteins, phenylpropanoid metabolism and flavonoid biosynthesis, reactive oxygen species (ROS) management, cell wall biosynthesis and remodeling, and defense regulation. The two genotypes exhibited distinct expression patterns of receptor and kinase genes. *Mitogen-activated protein kinase 1* (*MAPK1*) and *PTI1-like tyrosine-protein kinase 3* genes were upregulated exclusively in the resistant line SUT13E18305, indicating their potential role in initiating resistance-specific signaling cascades. In contrast, *MAPK kinase 3* (*MKK3*) and *chitin elicitor receptor kinase 1* (*CERK1*) were specifically upregulated in the susceptible line SUT16C007, suggesting divergent pathogen recognition mechanisms. The gene encoding protein P21-like, a potential cell cycle regulator or immune-related modulator, was consistently downregulated in both genotypes. All DEGs associated with hormone signaling and transcriptional regulation were downregulated in the susceptible genotype, indicating a compromised or delayed defense response. In contrast, these genes were largely unaltered in the resistant genotype, suggesting a more stable or pre-primed hormonal and transcriptional network under pathogen stress.

The differential expression of PR genes further highlighted genotype-specific responses. In the resistant genotype, only the *pathogenesis-related protein 1-like* (*PR1-like*) gene was induced, while *thaumatin-like protein 1* gene expression was reduced, implying selective activation of PR genes. In contrast, most PR genes were broadly downregulated in the susceptible genotype, indicating weakened activation of the defense mechanism. Genes related to phenylpropanoid and flavonoid pathways also displayed genotype-specific expression. Isoflavone 2′-hydroxylase, a key enzyme in flavonoid biosynthesis, was upregulated solely in the resistant genotype, potentially contributing to the production of antimicrobial compounds. Conversely, phenylalanine ammonia-lyase (PAL), the entry-point enzyme of the phenylpropanoid pathway, was upregulated only in the susceptible genotype, suggesting differential metabolic flux. Genes involved in oxidative stress responses were either downregulated in the susceptible genotype or remained unaltered in the resistant genotype; this finding indicates insufficient ROS detoxification capacity in susceptible plants during the early infection stage.

Cell wall-associated genes were clearly repressed in the susceptible genotype. Genes encoding fasciclin-like arabinogalactan protein 8 and pectate lyase were exclusively downregulated in SUT16C007, while the β-*glucosidase 1-like* gene was downregulated in both genotypes, which potentially compromised structural barriers and defense-associated remodeling in susceptible plants. Interestingly, genes encoding MLO-like proteins, typically associated with susceptibility and defense modulation, were differentially expressed between the two genotypes; this observation suggests their potential roles in genotype-specific defense signaling or susceptibility (Supplementary Table 2). Collectively, these transcriptomic profiles reveal that early defense in *Dendrobium* involves rapid and genotype-specific regulation of signaling networks, transcriptional activity, and defense-associated metabolic pathways, which may influence the plant’s resistance or susceptibility to *P. parasitica*.

### Later-stage transcriptomic responses reveal enhanced and genotype-specific defense activation

In the later stage of *P. parasitica* infection, the number of upregulated DEGs increased in both *Dendrobium* genotypes, reflecting a more extensive activation of defense-related pathways. However, the differential expression patterns between the resistant and susceptible lines continued to remain a critical aspect of genotype-specific responses. Several signaling components showed stronger or exclusive induction in the resistant genotype. Notably, genes encoding chitin elicitor-binding protein-like (CEBiP-like) and mitogen-activated protein kinase kinase 9-like (MAPKK9-like) were highly induced only in the resistant line, suggesting a continued or amplified perception of pathogen-associated molecular patterns (PAMPs). These findings build upon the data from the early response stage, where *CERK1* was upregulated in the susceptible genotype but not in the resistant one, highlighting temporal- and genotype-dependent differences in chitin-mediated recognition. Moreover, genes encoding mitogen-activated protein kinase 5 (MAPK5) and PTI1-like tyrosine-protein kinase were upregulated in both genotypes, but to a greater extent in the resistant line. This finding is in contrast with the early-stage upregulation of genes encoding MAPK1 and PTI1-like kinase 3 in the resistant line alone, suggesting broader MAPK cascade activation over time.

The gene encoding ENHANCED DISEASE RESISTANCE 2-like (EDR2-like), a negative regulator of cell death, was more highly induced in the resistant genotype, indicating a tightly controlled immune response. The *NDR1/HIN1-like protein 3* gene, involved in effector-triggered immunity (ETI), was also upregulated in both genotypes, suggesting the activation of intracellular immune surveillance. Hormone signaling pathways showed sustained involvement in the defense response. Genes associated with JA and ethylene signaling, *jasmonoyl-L-amino acid synthetase JAR4* and *ethylene receptor 2*, were upregulated in both genotypes, suggesting coordinated hormone-mediated defense, albeit with generally higher transcript levels in the resistant line. Several TFs were prominently upregulated in the later stage. Genes encoding WRKY TFs 43, 53, 57, and 75 as well as ethylene-responsive transcription factor 4-like (ERF4-like), were induced in both genotypes, although their expression levels were consistently higher in the resistant line. This pattern resembles observations in the early infection stage, where WRKY family members were differentially regulated, establishing their role in orchestrating time-dependent immune responses. Genes encoding defense-related enzymes, such as chitinase, polyphenol oxidase (PPO), and peroxidase 51-like, were induced in both genotypes, but with markedly higher expression in the resistant line. This enhanced enzymatic activity may contribute to pathogen inhibition and cellular detoxification. Conversely, the *PR1-like* gene, which was specifically upregulated in the early infection stage in the resistant genotype, was downregulated in both genotypes in the later stage, suggesting its restricted role in early defense response. *Premnaspirodiene oxygenase-like*, a gene potentially involved in specialized metabolite biosynthesis, was upregulated in both genotypes but showed higher expression levels in the resistant line, indicating its potential contribution to the production of antimicrobial compounds. The *DETOXIFICATION 27 protein* gene was also upregulated in both genotypes, reflecting a broad response to pathogen-induced oxidative or chemical stress. Genes involved in cell wall dynamics exhibited divergent expression patterns in both genotypes. *Pectinesterase* and β-*glucosidase 11-like* were upregulated in both genotypes, with higher expression levels in the susceptible line, possibly reflecting a compensatory response to cell wall degradation. In contrast, the *xyloglucan endotransglucosylase/hydrolase protein 22* (*XTH22*) gene was exclusively induced in the resistant genotype, suggesting resistance-specific strengthening of the cell wall. Genes encoding EG45-like domain-containing protein and programmed cell death protein 4-like were specifically upregulated in the resistant genotype, suggesting their roles in HR and localized cell death. Similarly, the specific upregulation of the *probable flavin-containing monooxygenase 1* gene in the resistant line implies the activation of SAR through N-hydroxypipecolic acid biosynthesis (Supplementary Table 3).

Overall, the later stage of infection revealed a more robust and complex activation of defense networks, with the resistant genotype displaying enhanced expression of key genes involved in recognition, signaling, transcriptional regulation, and defense execution. Many of these DEGs were associated with pathways initiated in the early response phase, such as MAPK signaling, WRKY TFs, and PR gene regulation, highlighting a dynamic and genotype-specific orchestration of layered immune responses against *P. parasitica*.

Sixteen candidate genes associated with black rot resistance in *Dendrobium* were identified by analyzing significant DEGs in susceptible SUT16C007 and resistant SUT13E18305 genotypes at early (0 hpi vs. 12 hpi) and later (12 hpi vs. 24 hpi) infection stages (Table 3). The gene encoding PR1-like was upregulated in both genotypes during early infection but was downregulated in the later stage. The *Apoptosis inhibitor 5-like protein* gene was induced in both genotypes in the early infection stage. In contrast, the *phytosulfokine receptor 1* (*PSKR1*) gene was specifically downregulated in the susceptible line during early infection. Conversely, in the later infection stage, the resistant line showed unique upregulation of the *CEBiP-like* gene, while the susceptible line exhibited specific upregulation of the *signal recognition particle receptor* gene. Other candidate genes were upregulated in the later infection stage, except for *beta-glucosidase 11-like*, which was exclusively upregulated in the resistant line during early infection. During later infection, this gene was further upregulated in both resistant and susceptible lines, with a notably higher induction in the resistant line. At this time point, most defense-related genes exhibited greater fold changes in expression in the resistant line compared to those in the susceptible counterpart. These genes included *chitinase 2-like, peroxidase 51-like, PPO, WRKY transcription factors 43* and *75*, and *EDR2-like*, suggesting a more robust and coordinated defense response. Conversely, the susceptible line displayed higher expression fold changes for several genes associated with stress signaling and cell wall modification, such as *transcription repressor MYB6, jasmonoyl-L-amino acid synthetase JAR4, pectinesterase*, and *beta-glucosidase 11-like* (Table 3), potentially reflecting an inadequate or misregulated defense strategy.

**Table 3:** Gene expression of candidate genes associated with disease resistance mechanisms against *Phytophthora parasitica* at different time points post-inoculation in susceptible and resistant *Dendrobium* genotypes

No.	Gene ID	Description	Gene expression (fold change)							
			SUT16C007				SUT13E18305			
			(0 hpi vs. 12 hpi)		(12 hpi vs. 24 hpi)		(0 hpi vs. 12 hpi)		(12 hpi vs. 24 hpi)	
	Pathogen recognition and signal transduction									
1	LOC110098552	Chitin elicitor-binding protein-like	–	NS	–	NS	–	NS	24.6	NS
2	LOC110109042	Signal recognition particle receptor	–	NS	2.71	Up	–	NS	–	NS
	Pathogenesis-related (PR) proteins									
3	LOC110103682	Chitinase 2-like	–	NS	12.21	Up	–	NS	27.58	Up
4	LOC110097445	Peroxidase 51-like	–	NS	11.2	Up	–	NS	23.79	Up
5	LOC110100715	Pathogenesis-related protein 1-like	6.18	Up	0.09	Down	17.22	Up	0.09	Down
6	LOC110116766	Polyphenol oxidase	–	NS	105.54	Up	–	NS	159.7	Up
	Transcription factors									
7	LOC110091865	Probable WRKY transcription factor 43	–	NS	36.94	Up	–	NS	72.19	Up
8	LOC110104704	Probable WRKY transcription factor 75	–	NS	25.14	Up	–	NS	49.25	Up
9	LOC110111286	Transcription repressor MYB6	–	NS	57.62	Up	–	NS	36.95	Up
	Hormone signaling pathway related genes									
10	LOC110100668	Jasmonoyl-L-amino acid synthetase JAR4	–	NS	4.04	Up	–	NS	2.69	Up
11	LOC110108710	Protein ENHANCED DISEASE RESISTANCE 2-like	–	NS	10.94	Up	–	NS	55.6	Up
12	LOC110104800	Phytosulfokine receptor 1	0.37	DOWN	–	NS	–	NS	–	NS
	Phenylpropanoid metabolism									
13	LOC110115785	Phenylalanine ammonia-lyase-like	–	NS	2.72	Up	–	NS	–	NS
	Hypersensitive response									
14	LOC110102591	Apoptosis inhibitor 5-like protein	2.94	Up	–	NS	2.46	Up	–	NS
	Cell wall reinforcement and modulation									
15	LOC110115516	Pectinesterase	–	NS	463.48	Up	–	NS	35.49	Up
16	LOC110113000	Beta-glucosidase 11-like	–	NS	332.71	Up	7.58	Up	27.46	Up

### Gene expression analysis through qPCR

**Figure 6:**
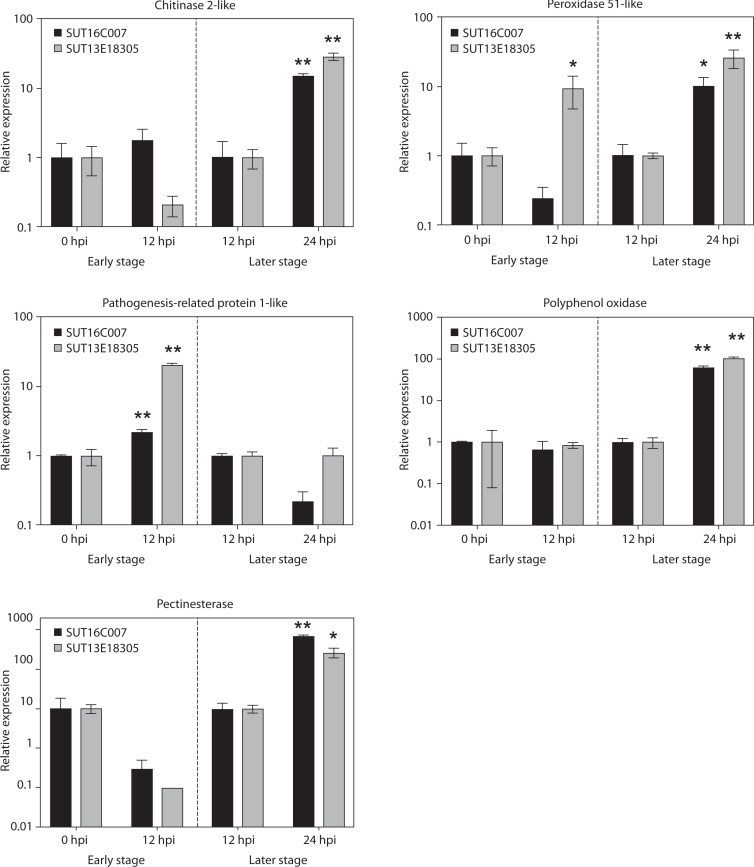
Expression analysis of putative genes involved in black rot resistance in Dendrobium “Earsakul” by qPCR. The y-axis indicates the relative expression of Phytophthora parasitica inoculated at 0-, 12-, and 24-hpi. The X-axis represents the susceptible variety SUT16C007 and resistant line SUT13E18305 in the early and later infection stages. * and ** indicate significant difference in the early infection stage (0 hpi vs. 12 hpi) and the later infection stage (12 hpi vs. 24 hpi) of each line; n = 3, p < 0.05 and 0.01, respectively

Among the 16 DEGs, we selected 8 DEGs to validate gene expression patterns through qPCR, with actin as the endogenous control. Although the RNA-seq and qPCR data exhibited slight variations in fold change values (Figure 6), the overall trends remained consistent.

The *PR1-like* gene was highly upregulated in both genotypes in the early infection stage (*p* < 0.01), with a stronger induction in the resistant line (20.44-fold) compared to that in the susceptible line (2.21-fold). However, this gene showed no significant differential expression at 24 hpi. In contrast, genes associated with cell wall biosynthesis (*pectinesterase*) and other PR proteins (*chitinase 2-like* and *PPO*) were specifically upregulated in both genotypes in the later infection stage (*p* < 0.05; *p* < 0.01) but not during early infection. The susceptible line showed 68.42-, 14.48-, and 63.10-fold induction of these genes, respectively, whereas the resistant line displayed 25.80-, 28.32-, and 103.19-fold upregulation of these genes, respectively. Additionally, the gene encoding peroxidase 51-like was significantly upregulated (9.29-fold) exclusively in the resistant line in the early infection stage. In contrast, this gene showed moderate upregulation (10.08-fold, *p* < 0.05) in the susceptible line and a much stronger induction (25.74-fold) in the resistant line in the later infection stage.

Finally, the genes encoding CEBiP-like, probable WRKY transcription factor 43, and probable WRKY transcription factor 75 could not be amplified.

## Discussion

Infection with *Phytophthora* species causes substantial economic losses to global agricultural and ornamental crop production (Yu et al. 2020). *P. parasitica* is a hemibiotrophic, destructive oomycete pathogen, with an initial biotrophic phase; it derives nutrients from living host tissues, before transitioning to a necrotrophic phase, where they kill host cells to obtain nutrients. Infection due to this pathogen adversely affects the quantitative and qualitative yield of many plant species, including *Dendrobium* orchids. Hence, elucidating the molecular and genetic determinants of plant resistance mechanisms against these pathogens is critical to develop effective disease control strategies. RNA-Seq analysis is a high-throughput technology for investigating the gene expression levels under various conditions (Kukurba and Montgomery 2015). In previous studies, RNA-Seq analysis has been used to identify the DEGs associated with disease resistance mechanisms in tobacco, soybean, potato, and durian (Duan et al. 2020; Meng et al. 2021; Li et al. 2023; Nawae et al. 2024). In the present study, we conducted comparative transcriptomic analysis between the resistant SUT13E18305 and susceptible SUT16C007 *Dendrobium* “Earsakul” lines subjected to *P. parasitica* challenge to obtain profound insights into the molecular mechanisms underlying black rot resistance. The phenotypic evaluation demonstrated clear divergence between the resistant and susceptible lines. While both genotypes exhibited similar disease severity scores at early time points (0–3 DAI), significant differences emerged by 5 DAI, with the resistant line showing minimal symptoms (HR classification), and the susceptible line progressing to moderate (MS) and eventually to susceptible (S) classification by 7 DAI. The resistant line showed much slower progression of the symptoms, maintaining its resistant (R) status even at 7 DAI. This delayed onset of symptoms suggests that the resistant line employs both inducible defense mechanisms and constitutive barriers. The lack of early symptoms in the resistant line aligns with the concept of ETI where recognition of pathogen effectors activates robust defense responses (Figure 2).

RNA-Seq analysis revealed dynamic transcriptional changes in both genotypes. The susceptible line displayed a larger number of DEGs than the resistant line; however, the resistant line exhibited a more targeted and sustained defense response (Figure 3). Interestingly, GO analysis revealed that the DEGs involved in resistance mechanism-related GO terms were more predominant in the resistant line, and most of these genes were upregulated (Figure 4). Moreover, the KEGG pathway enrichment analysis revealed diverse gene functions, demonstrating that DEGs associated with resistance mechanisms were enriched in both *Dendrobium* genotypes (Figure 5). These findings delineate a multifaceted defense strategy involving pathogen recognition, signal transduction, hormone signaling pathway, and HR for defense in the early biotrophic phase. In contrast, the genes involved in PR proteins and antimicrobial synthesis, ROS management, and cell wall strengthening for defense response in the later response phase collectively contribute to the resistant phenotype. However, the potential for false negatives in RNA-Seq analysis necessitates validation of transcript expression. In this study, qPCR analysis of 5 of 8 candidate genes confirmed the RNA-Seq data (Figure 6). This similar expression result suggests a consistent expression pattern for the validated upregulated and downregulated genes in RNA-Seq and qPCR analyses. The failure of the remaining three genes to amplify may be attributed to low cDNA concentration or inefficient primers. Future studies to validate these transcripts should utilize higher cDNA input or newly designed primer sets.

### Pathogen recognition and early defense activation

In the early infection stage, *Dendrobium* regulated the genes involved in pathogen recognition, hormone signaling, ROS management, and HR for defense in the biotrophic phase. Subsequently, the genes involved in cell wall remodeling and antimicrobial production and defense responses were induced. The plant immune response is triggered by the perception of evolutionarily conserved microbial signatures, termed microbe- or pathogen-associated molecular patterns (MAMPs/PAMPs). Receptors play a crucial role in pathogen recognition, initiating downstream signaling cascades that lead to defense responses. The gene encoding signal recognition particle receptor subunit alpha homolog (LOC110109042) was upregulated in the susceptible genotype (2.71-fold) in the early infection stage. While primarily involved in co-translational protein targeting to the endoplasmic reticulum or chloroplasts (Keenan et al. 2001), proper protein localization is essential for the function of many defense-related proteins, including pattern recognition receptors (PRRs) and receptor-like kinases (RLKs). The upregulation of this component, even in the susceptible genotype, might reflect an attempt to increase the synthesis and proper delivery of membrane-bound defense receptors or other proteins involved in cellular communication during the initial perception of PAMPs, which is an essential component of PAMP-triggered immunity (PTI) (Jones and Dangl 2006; Monaghan and Zipfel 2012). This finding suggests an early recognition attempt, even if the downstream immune response is ultimately insufficient in the susceptible *Dendrobium* line.

This recognition occurs through plasma membrane-localized PRRs, predominantly comprising RLKs and receptor-like proteins, which subsequently activate the basic defense response known as PTI (Jones and Dangl 2006; Monaghan and Zipfel 2012). The CEBiP-like receptor probably plays a role in early pathogen recognition. CEBiPs are plant cell surface receptors that recognize chitin fragments (chito-oligosaccharides) released from fungal cell walls. This recognition is crucial to perceive fungal pathogens and trigger downstream signaling pathways that lead to plant immune responses (Kaku et al. 2006). Its differential expression patterns between the genotypes can facilitate elucidation of its precise role in the recognition and subsequent activation of plant defense. The resistant line exhibited rapid upregulation of CEBiP (LOC110098552) and RLKs, which are critical for recognizing PAMPs. CEBiP binds to chitin oligosaccharides, thereby triggering PTI (Reyes Zamora et al. 2024). The absence of CEBiP induction in the susceptible line suggests impaired PTI, rendering it vulnerable to *P. parasitica*. This finding aligns with studies on *Arabidopsis*, where RLK-deficient mutants display increased susceptibility to oomycetes (Monaghan and Zipfel 2012). The second level involves plant R proteins, which are the key components of plant immunity, often recognizing specific pathogen effectors and triggering strong defense responses, including ETI (Jones and Dangl 2006). The gene encoding EDR2-like protein (LOC110108710) showed a prominent increase in expression in both genotypes, particularly in the resistant genotype, highlighting its potential role in race-specific resistance and ETI. This gene induced SA-mediated responses and stimulated resistance to the biotrophic pathogen, leading to the activation of programmed cell death (PCD) and SAR (Tang et al. 2005; Vorwerk et al. 2007).

At 12 hpi, *PR-1* expression was upregulated in both genotypes, with a moderate increase observed in SUT16C007 (6.18-fold) and a markedly stronger induction in SUT13E18305 (17.22-fold). This finding suggests a more robust early defense response in the resistant genotype. However, by 24 hpi, *PR-1* expression levels declined in both genotypes, indicating a transient activation of this PR gene following pathogen challenge. PR-1 proteins are commonly considered hallmark genes of the SA-mediated defense pathway, which is essential for resistance against biotrophic and hemibiotrophic pathogens such as *P. parasitica* (van Loon et al. 2006; Fu and Dong 2013). Although the precise biochemical function of PR-1 proteins remains controversial, they are known to possess antifungal activity and are thought to contribute to broad-spectrum disease resistance (van Loon et al. 2006). In this study, the early and strong induction of PR-1, particularly in the resistant genotype, suggests rapid activation of the SA-dependent defense pathway (Uknes et al. 1993; Fu and Dong 2013). This early response is crucial for initial pathogen recognition or signal initiation, as the SA pathway often orchestrates the transcriptional reprogramming necessary for an effective defense response. The subsequent downregulation could indicate a transient peak in expression, or a shift in the defense strategy as the plant progresses through the infection course, fine-tuning its response to limit resource expenditure or avoid detrimental prolonged immune activation.

### Transcriptional reprogramming

Signal transduction leads to transcriptional reprogramming and defense-related gene expression. TFs are crucial regulatory proteins that bind to specific DNA sequences to control gene expression, thus orchestrating complex plant defense responses. WRKY TFs regulate a wide array of defense-related genes. Interestingly, two TFs, WRKY43 (LOC110091865) and WRKY75 (LOC110104704), exhibited upregulation in the later infection stage (24 hpi), particularly in the resistant genotype (SUT13E18305). Their expression patterns indicate an activation of secondary immune signaling cascades that amplify and sustain the defense response. WRKY TFs constitute a large plant-specific family that plays a central role in regulating various plant processes, including development, senescence, and responses to biotic and abiotic stresses. Many WRKYs act as positive regulators of plant immunity by activating defense-related genes through binding to W-box *cis*-elements in their promoters (Rushton et al. 2010; Phukan et al. 2016). Specifically, WRKY43 and WRKY75 have been implicated in mediating stress responses and functioning as positive regulators of disease resistance, linked to the SA pathway and PCD (Miao and Zentgraf 2007; Yao et al. 2012). The significant upregulation of both WRKY43 and WRKY75 in the resistant genotype suggests their crucial role in initiating and sustaining robust defense responses against *P. parasitica*. MYB TFs are another large family of TFs involved in diverse plant processes, including secondary metabolism and stress responses (Dubos et al. 2010). To extract nutrients from host cells, hemibiotrophic pathogens may employ certain effectors to maintain plant cell viability during infection. Ma et al. (2021) reported that MYB6 competes with the effector of *Verticillium dahlia* (*Verticillium dahliae*-secreted Asp f2-like protein VDAL, which causes leaf wilting) to bind to plant U-box 25 (PUB25) and PUB26. The increased accumulation of MYB6 improved disease resistance in *Arabidopsis*. In this study, the gene encoding transcription repressor MYB6 was upregulated in both susceptible and resistant lines, suggesting that it may participate in early defense signaling or general stress responses. However, its presence in both genotypes implies that MYB6 alone may not be a determinant of resistance but could play a modulatory role within broader defense pathways.

### Hypersensitive response

HR is a form of PCD at the infection site, which limits pathogen spread and is a hallmark of effective plant resistance (Lam et al. 2001). The apoptosis inhibitor 5-like protein (LOC110102591) exhibited transient upregulation in both susceptible and resistant genotypes, suggesting its role in modulating PCD during early infection. The expression of this protein may contribute to balance cell death and survival, which is critical for preventing excessive tissue damage. Although present in both genotypes, its regulation in the resistant line may support a more controlled HR, whereas this response may be less effective in the susceptible line. Further studies are required to clarify its functional significance in resistance pathways.

### Hormone signaling pathway and activation of pathogenesis-related proteins

Phytosulfokine receptor 1 (PSKR1) was downregulated in the susceptible genotype but remained stable in the resistant line. PSKR1 can negatively regulate SA-dependent defense responses (Segonzac and Monaghan 2019), suggesting that its downregulation in the susceptible line may reflect a deregulated defense signaling network. PSKR1 is also implicated in hormonal crosstalk, particularly in modulating JA signaling, which plays a key role in resistance to necrotrophic pathogens. Interestingly, the gene encoding jasmonoyl-L-amino acid synthetase (LOC110100668), a key enzyme in JA biosynthesis, was upregulated in both genotypes at 24 hpi, indicating the activation of the JA pathway in response to *P. parasitica* infection (Ruan et al. 2019). These findings suggest that, although both *Dendrobium* genotypes initiated JA-mediated defenses, the differential regulation of PSKR1 may influence the balance between SA- and JA-mediated responses, potentially contributing to the distinct resistance outcomes.

In the later infection stage, many defense genes were induced, including the gene involved in PR proteins and antimicrobial compound synthesis, ROS management, cell wall remodeling, and phenylpropanoid metabolism. PR proteins are a diverse group of plant proteins that are induced upon pathogen attack and play crucial roles in various aspects of plant defense. Their upregulation is a hallmark of plant immune responses and is often associated with SAR and localized defense responses. Genes encoding chitinase 2-like proteins, such as LOC110103682, showed a significant upregulation in the resistant line (SUT13E18305) (27.58-fold) at 24 hpi, whereas it was less induced in the susceptible genotype (SUT16C007) (12.21-fold). The stronger and potentially earlier induction in the resistant line highlights the importance of this gene in mounting an effective defense against *P. parasitica*. Chitinases (classified as PR-3, PR-4, PR-8, and PR-11 families) are hydrolytic enzymes that specifically degrade chitin, a major structural component of fungal and oomycete cell walls (though oomycetes primarily contain glucans, they also have chitin in some stages or structures, and chitinases can also act on modified glucans or have broader antimicrobial activity) (Grover and Gowthaman 2009; Nawrot and Błaszczyk 2020). By degrading chitin, these enzymes directly inhibit pathogen growth and release chitin fragments (chito-oligosaccharides) that act as PAMPs (Shibuya and Minami 2001). These PAMPs serve as elicitors, triggering downstream defense signaling pathways in plants, including the activation of an oxidative burst and the induction of other defense genes. The observed upregulation in the later infection stage suggests their sustained role in directly combating the invading pathogen by compromising its structural integrity.

The expression pattern of the gene encoding peroxidase 51-like protein (LOC110097445) closely mirrored that of chitinase, with significantly higher induction observed in the resistant genotype at 24 hpi (23.79-fold) compared to that in the susceptible genotype (11.2-fold). This differential expression suggests a general role of peroxidase in defense response against pathogens, but with a more robust and potentially more effective deployment in the resistant genotype. Peroxidases, classified as PR-9 proteins, are heme-containing enzymes that contribute to various defense processes, notably ROS management and strengthening of plant cell walls (Passardi et al. 2005; Almagro et al. 2009). Their upregulation aligns with their known role in contributing to oxidative burst, an early defense response characterized by a rapid accumulation of ROS such as hydrogen peroxide (H_2_O_2_) (Apel and Hirt 2004). ROS not only exert direct antimicrobial effects but also act as signaling molecules to activate downstream defense pathways and promote the cross-linking of cell wall polymers (e.g., lignification and suberization), thereby enhancing physical barriers to pathogen invasion. The sustained induction of LOC110097445 in the resistant genotype reveals its dual role in both immediate and prolonged defense responses, contributing to a more effective resistance phenotype.

PPO (LOC110116766) was strongly upregulated, particularly in the resistant genotype (SUT13E18305), suggesting its role in resistance against *P. parasitica*. PPOs are copper-containing enzymes that catalyze the oxidation of phenolic compounds to quinones, which are highly reactive. This process, often observed as browning, is associated with wound healing and defense responses, as quinones can directly inhibit pathogen enzymes, cross-link with proteins to make them less digestible for pathogens, and contribute to the production of antimicrobial substances (Thipyapong et al. 2004; Constabel and Barbehenn 2008). The prominent upregulation in the resistant genotype indicates robust activation of this defense mechanism.

### Cell wall remodeling and modulation

Genes associated with cell wall modification exhibited differential expression patterns that likely contribute to defense responses against *P. parasitica*. In the resistant genotype (SUT13E18305), the β-*glucosidase 11-like* gene (LOC110113000) was upregulated early at 12 hpi (7.58-fold), and its expression was further increased at 24 hpi (27.46-fold), suggesting a rapid and sustained chemical defense response. In contrast, the susceptible genotype (SUT16C007) showed a delayed but markedly higher induction at 24 hpi (332.71-fold), indicating late defense activation that may be insufficient for effective resistance. β-Glucosidase 11-like, a member of glycoside hydrolase family 1, plays a role in plant defense by cleaving β-glycosidic linkages in cell wall-associated glucosides. This enzymatic activity releases lignin precursors and signaling compounds, facilitating cell wall strengthening and structural modification to improve the plant’s physical defense against pathogen entry (Xu et al. 2024). β-Glucosidases could also release active defense compounds such as cyanogenic glucosides or glucosinolates, which can generate toxic aglycones upon hydrolysis (Morant et al. 2008). Their early induction in the resistant line highlights their role in the rapid mobilization of chemical defenses. Similarly, the gene encoding pectinesterase (PEs) (LOC110115516) was upregulated in both genotypes at 24 hpi, indicating a role in cell wall remodeling during infection. PEs catalyze the de-esterification of pectin, a key structural polysaccharide in the plant cell wall, thereby influencing cell wall rigidity and porosity (Rihouey et al. 2017). While some PEs can be exploited by pathogens to facilitate invasion, plant-derived PEs may also contribute to defense by restructuring cell wall barriers or by generating pectin-derived oligogalacturonides that act as damage-associated molecular patterns. The consistent upregulation of PE in both genotypes suggests a conserved, general defense mechanism involving regulated cell wall modification.

### Phenylpropanoid metabolism

The phenylpropanoid pathway is a central secondary metabolic route in plants, producing a wide range of compounds with critical roles in structural reinforcement and defense. In this study, the PAL-like gene (LOC110115785), a key enzyme catalyzing the entry step into the phenylpropanoid pathway (Hahlbrock and Scheel 1989), was upregulated at 24 hpi in the susceptible genotype (2.72-fold), but not in the resistant genotype. PAL has a role in the biosynthesis of lignin, flavonoids, and phytoalexins, which contribute to cell wall fortification and antimicrobial activity (Dixon and Paiva 1995). The induction of *PAL* in the susceptible line likely reflects an attempt to strengthen physical barriers and mount chemical defenses in response to *P. parasitica* infection. However, the timing and relatively modest level of induction suggest a delayed or insufficient response, which may be inadequate to halt pathogen progression.

Overall, the resistant *Dendrobium* “Earsakul” genotype exhibited a more timely and coordinated activation of defense responses. This included pathogen recognition, rapid signal transduction mediated by WRKY and MYB TFs, and activation of multiple defense layers such as SA and JA signaling, ROS generation, HR, and PR protein accumulation. Although many defense-related genes were also upregulated in the susceptible genotype, the responses were generally delayed or attenuated, suggesting that the temporal dynamics and magnitude of defense activation are the critical determinants of resistance effectiveness.

## Conclusions

This study presents a comprehensive transcriptomics analysis of black rot resistance in *Dendrobium* “Earsakul” by comparing resistant (SUT13E18305) and susceptible (SUT16C007) genotypes in response to *P. parasitica* infection. The findings reveal that resistance to this pathogen is mediated by a complex and coordinated defense network involving pathogen recognition, activation of signal transduction pathways, and extensive transcriptional reprogramming. The resistant genotype exhibited timely induction of genes associated with SA and JA signaling, enhanced expression of TFs (e.g., WRKY and MYB), and modulation of hormonal crosstalk, contributing to effective immune activation. Key defense-related genes involved in PR protein accumulation, antimicrobial compound biosynthesis, ROS generation and detoxification, cell wall remodeling, and phenylpropanoid metabolism were also differentially expressed. These components function synergistically through interconnected signaling cascades to strengthen both structural and biochemical barriers, ultimately enhancing resistance to *P. parasitica*. Future studies employing functional genomics approaches, such as gene knockout, overexpression, or CRISPR-based validation, are crucial for confirming the roles proposed in this study and to accelerate the development of black rot-resistant orchids and support sustainable ornamental crop production.

## Supplementary Material







## Data Availability

The datasets presented in this study can be found in online repositories. The name of the repository and accession number can be found below: NCBI BioProject repository under the accession PRJNA1345543. All data and materials included in this study are available upon request from the corresponding author.
