# Supplementary material for: Comparative RNA sequencing analysis of resistant and susceptible Dendrobium “Earsakul” under black rot challenge
Source: BioTechnologia (Pozn). 2026 Mar 25;107(1):23–44. doi: 10.5114/bta/216300 (PMC13127364; doi:10.5114/bta/216300)
Supplement: Supplementary file 1 [file BTA-107-1-216300-s1.pdf]

Supplementary Table 1. Primer details for qPCR validation

Target	Primer sequence (5' ---> 3')	Tm (C°)
Chitinase	F: GCAGGGACATTACCAGGCTT<br>R: CTTCCATCGGTGGTGAAGCT	60
Peroxidase 51-like	F: GTTTCATTACAGCGGCCCAC<br>R: TGTTCGGTTCAAGCTTGGGT	60
Pathogenesis-related protein 1-like	F: TCTGTTGTGGAGGGTGGAGA<br>R: GTTCCAGGATTGGCTGCAAG	60
Polyphenol oxidase	F: GGTTCCTGCGTTCCCCATTA<br>R: TCCACCAAAAGCACCTCCTC	60
Pectinesterase	F: CTTCAGTCGTCGACGGTGAA<br>R: CATCGACGACGTTCTGGCTA	60
Actin (reference gene)	F: AGTTCCTATTTATGAGGGTTATGC<br>R: CCTGACAATTTCACGCTCTG	60
