# Supplementary material for: Comparative RNA sequencing analysis of resistant and susceptible Dendrobium “Earsakul” under black rot challenge
Source: BioTechnologia (Pozn). 2026 Mar 25;107(1):23–44. doi: 10.5114/bta/216300 (PMC13127364; doi:10.5114/bta/216300)
Supplement: Supplementary file 2 [file BTA-107-1-216300-s2.pdf]

Supplementary Table 2. Genes differentially expressed in the resistant line SUT13E18305 and susceptible line SUT16C007 at 0 hpi compared to 12 hpi in response to *P. parasitica* infection causing black rot disease.

Gene ID	Description	Log_2 fold change	
		SUT13E18305	SUT16C007
**Pathogen recognition and signal transduction**			
LOC110096388	chitin elicitor receptor kinase 1	ns	1.13
LOC110092819	protein P21-like	-3.87	-4.36
LOC110112011	mitogen-activated protein kinase 1	1.40	ns
LOC110105770	mitogen-activated protein kinase kinase 3	ns	1.40
LOC110105312	PTI1-like tyrosine-protein kinase 3	1.53	ns
**Hormone signaling pathway-related genes**			
LOC110102787	NDR1/HIN1-like protein 13	-1.69	-2.27
LOC110106032	protein EDS1	-1.44	-1.21
LOC110108011	probable linoleate 9S-lipoxygenase 5	ns	-1.58
LOC110104800	phytosulfokine receptor 1	ns	-1.45
**Transcription factors**			
LOC110099322	probable WRKY transcription factor 2	ns	-2.16
LOC110099934	probable WRKY transcription factor 31	ns	-3.17
LOC110111979	probable WRKY transcription factor 40	ns	-1.39
LOC110114132	probable WRKY transcription factor 48	ns	-2.27
LOC110103316	probable WRKY transcription factor 50	ns	-1.63
LOC110092784	probable WRKY transcription factor 57	ns	-2.05
LOC110094940	probable WRKY transcription factor 65	ns	-5.63
LOC110098909	transcription factor bHLH104	-3.79	-4.24
LOC110099272	transcription factor MYB2-like	ns	-4.63
LOC110103484	transcription factor MYBS3	ns	-5.24
LOC110109092	pathogenesis-related genes transcriptional activator PTI5-like	ns	-3.74
LOC110099586	pathogenesis-related genes transcriptional activator PTI6	ns	-1.73
**Pathogenesis-related proteins**			
LOC110100703	pathogenesis-related protein 1	ns	-1.44
LOC110100715	pathogenesis-related protein 1-like	4.11	ns
LOC110098653	peroxidase 4	ns	-4.37
LOC110094241	proteinase inhibitor PSI-1.2	ns	-2.72
LOC110097861	thaumatin-like protein 1	-3.90	-2.16
**Phenylpropanoid metabolism and flavonoid biosynthesis**			
LOC110115676	isoflavone 2'-hydroxylase	6.38	ns
LOC110113904	phenylalanine ammonia-lyase	ns	1.09
**ROS-related proteins**			
LOC110111028	superoxide dismutase [Cu-Zn]	ns	-1.64
LOC110103395	glutathione S-transferase U24-like	ns	-2.31
LOC110099103	NADH dehydrogenase [ubiquinone] 1	ns	-3.23

Gene ID	Description	Log$_2$ fold change	
		SUT13E18305	SUT16C007
**Cell wall biosynthesis and modulation**			
LOC110092839	β-glucosidase 1-like	-3.03	-5.09
LOC110112015	cell division protein FtsY homolog	1.03	ns
LOC110104487	fasciclin-like arabinogalactan protein 8	ns	-3.65
LOC110115929	pectate lyase	ns	-1.40
**Defense regulation**			
LOC110097882	MLO-like protein 1	ns	2.34
LOC110093886	MLO-like protein 9	ns	-1.81
LOC110107652	MLO-like protein 13	-1.08	-1.77
LOC110099817	MLO-like protein 14	-2.84	ns
