# Supplementary material for: Comparative RNA sequencing analysis of resistant and susceptible Dendrobium “Earsakul” under black rot challenge
Source: BioTechnologia (Pozn). 2026 Mar 25;107(1):23–44. doi: 10.5114/bta/216300 (PMC13127364; doi:10.5114/bta/216300)
Supplement: Supplementary file 3 [file BTA-107-1-216300-s3.pdf]

Supplementary Table 3. Genes differentially expressed in the resistant line SUT13E18305 and susceptible line SUT16C007 at 12 hpi compared to 24 hpi in response to *P. parasitica* infection causing black rot disease.

Gene ID	Description	Log_2 fold change	
		SUT13E18305	SUT16C007
**Pathogen recognition and signal transduction**			
LOC110098552	chitin elicitor-binding protein-like	4.62	ns
LOC110092500	mitogen-activated protein kinase 5	1.42	2.01
LOC110097015	mitogen-activated protein kinase kinase 9-like	3.81	ns
LOC110110173	PTI1-like tyrosine-protein kinase	1.57	2.40
**Hormone signaling pathway-related genes**			
LOC110095681	NDR1/HIN1-like protein 3	1.74	2.69
LOC110108710	protein ENHANCED DISEASE RESISTANCE 2-like	5.80	3.45
LOC110100668	jasmonoyl-L-amino acid synthetase JAR4	1.43	2.02
LOC110104881	ethylene receptor 2	1.12	1.34
**Transcription factors**			
LOC110091865	probable WRKY transcription factor 43	6.17	5.21
LOC110093969	probable WRKY transcription factor 53	5.26	3.00
LOC110109443	probable WRKY transcription factor 57	3.50	2.45
LOC110104704	probable WRKY transcription factor 75	5.62	4.65
LOC110096335	ethylene-responsive transcription factor 4-like	3.37	1.34
**Pathogenesis-related proteins**			
LOC110100715	pathogenesis-related protein 1-like	-3.49	-3.53
LOC110103682	chitinase 2-like	4.79	3.61
LOC110116766	polyphenol oxidase	7.32	6.72
LOC110097445	peroxidase 51-like	4.57	3.49
**Phenylpropanoid metabolism and flavonoid biosynthesis**			
LOC110098469	premnaspirodiene oxygenase-like	4.66	3.48
**ROS-related proteins and detoxification**			
LOC110098802	protein DETOXIFICATION 27	1.08	1.55
**Cell wall biosynthesis**			
LOC110098232	xyloglucan endotransglucosylase/hydrolase protein 22	1.53	ns
LOC110115516	pectinesterase	5.15	8.86
LOC110113000	β-glucosidase 11-like	4.78	8.38
**Hypersensitive response**			
LOC110095201	programmed cell death protein 4-like	1.15	ns
LOC110113333	EG45-like domain containing protein	6.07	ns
**Systemic acquired resistance**			
LOC110113621	probable flavin-containing monooxygenase 1	5.28	ns
